# A comprehensive longitudinal analysis of the cellular immune response specific to the spike protein in healthcare workers vaccinated against SARS-CoV-2– ORCHESTRA Project

**DOI:** 10.3389/fimmu.2025.1707449

**Published:** 2025-11-25

**Authors:** Stefano Ugel, Akshita Gupta, Gianluca Spiteri, Pierpaolo Marchetti, Francesco De Sanctis, Shari Wouters, Angelina Konnova, Maria Grazia Lourdes Monaco, Angela Carta, Maria Diletta Pezzani, Filippo Liviero, Sofia Pavanello, Marco dell’Omo, Eleonóra Fabiánová, Jana Bérešová, Francesca Larese Filon, Marcella Mauro, Giuseppe Verlato, Vincenzo Bronte, Samir Kumar-Singh, Stefano Porru

**Affiliations:** 1 Section of Immunology, Department of Medicine, University of Verona, Verona, Italy; 2 Molecular Pathology Group, Cell Biology and Histology, Faculty of Medicine and Health Sciences and Laboratory of Medical Microbiology, Vaccine and Infectious Disease Institute, University of Antwerp, Antwerp, Belgium; 3 Occupational Medicine Unit, University Hospital of Verona, Verona, Italy; 4 Unit of Epidemiology and Medical Statistics, Department of Diagnostics and Public Health, University of Verona, Verona, Italy; 5 Section of Occupational Medicine, Department of Diagnostics and Public Health, University ofVerona, Verona, Italy; 6 Infectious Diseases, Department of Diagnostics and Public Health, University Hospital of Verona, Verona, Italy; 7 Department of Cardio-Thoracic-Vascular Sciences and Public Health, University of Padova, Padova, Italy; 8 Occupational Medicine Unit, University Hospital of Padova, Padova, Italy; 9 University Center for Space Studies and Activities “Giuseppe Colombo” - CISAS, University of Padua, Padua, Italy; 10 Unit of Occupational Medicine, Department of Medicine and Surgery, University of Perugia, Perugia, Italy; 11 Occupational Health Department, Regional Authority of Public Health (RAPH), Banská Bystrica, Slovakia; 12 Unit of Occupational Medicine, Department of Medical Science, University of Trieste, Trieste, Italy

**Keywords:** SARS-CoV-2 infection (COVID-19), mRNA vaccine against SARS-CoV-2, post-vaccination immunity, anti-spike T-cell response, cytotoxic T cells (CTLs)

## Abstract

**Introduction:**

The long-term dynamics of T-cell immunity following SARS-CoV-2 vaccination, essential for durable protection, remain incompletely understood. This study, therefore, aimed to investigate the kinetics and persistence of spike-specific T-cell responses in vaccinated healthcare workers.

**Methods:**

Within the framework of the ORCHESTRA Project, we conducted a longitudinal study on the kinetics and persistence of CD4+ and CD8+ T cell immunity in healthcare workers (n=305) from four hospitals and public health centers across two European countries who received either 2 or 3 doses of an mRNA vaccine, with or without prior SARS-CoV-2 infection. Specifically, the anti-spike adaptive immune cellular response was evaluated, focusing on its crosstalk with the B cell response as measured by serology. Circulating cellular adaptive immune cells were extensively analyzed using flow cytometry to assess pro-inflammatory cytokine production (TNF-α, IFN-γ, IL-2), functional activation (CD154), and memory differentiation (CD45RO).

**Results:**

Our findings show that anti-spike T cell reactivity is not influenced by age, with the only exception of a weak positive correlation with spike-specific CD8+CD45RO+ T lymphocytes (Spearman’s rho = 0.34, p<0.001), and an equally weak negative correlation with CD8+TNF+ (Spearman’s rho = -0.23, p<0.01). Other variables, such as gender and job category, did not significantly impact the vaccine-induced, anti-spike T cell immune response.

**Discussion:**

No distinct relationship between CD4+ and CD8+ T cell subsets was observed post-vaccination. However, specific dynamic changes in vaccine-induced T cells were identified showing clear dose- and time-dependence. Finally, the median level of CD8+CD154+ lymphocytes, indicative of activated T cells, was significantly associated with infection incidence and may represent a reliable predictive biomarker. This study provides evidence that the vaccine-induced anti-spike cellular immune response should be considered when making vaccination decisions, as it has predictive value for infection risk.

## Introduction

The mRNA-based vaccines have demonstrated strong efficacy in protecting the population from severe illnesses and fatalities associated with SARS-CoV-2 (1, 2). Despite extensive efforts to control the COVID-19 outbreak, evaluating individuals’ vaccination status is essential to improve global vaccination strategies (3). The immunity gained from natural infection or vaccination provides substantial protection against reinfection and the development of severe COVID-19 that may require hospitalization (4, 5). Current estimates suggest that approximately 90% of people who have recovered from a natural SARS-CoV-2 infection retain protective immunity for at least eight months. In comparison, vaccinated individuals experience a risk reduction ranging from 50% to 95% (6, 7). Depending on factors such as vaccine type, time since the last dose, age, immune competence, history of prior SARS-CoV-2 infection, and circulating viral variants (8–11).

The adaptive immune system, comprising B and T lymphocytes, plays a crucial role in managing SARS-CoV-2 infections, aiding in viral clearance and providing protection against reinfection and severe disease following vaccination (12–14). Indeed, vaccination against SARS-CoV-2 significantly enhances the generation of B cells that produce virus-specific antibodies (15). Actually, several studies have shown that mRNA vaccines, such as those from Pfizer-BioNTech and Moderna, stimulate strong B cell responses, producing high-affinity neutralizing antibodies (2, 16, 17). These vaccines promote the activation and proliferation of naïve B cells in the germinal centers, where they undergo somatic hypermutation and affinity maturation, resulting in the development of memory B cells and long-lived plasma cells that together contribute to durable immunity (18). This process is crucial for generating a robust antibody response that can protect against reinfection and severe outcomes. Antibody levels typically peak within a few weeks after the second dose and then gradually decline over time. Studies suggest that protective antibody levels can persist for at least 6 to 8 months, but the exact duration can vary based on several factors, including individual immune response, age, and prior exposure (18–20). Although the quantity of circulating antibodies may decrease over time, memory B cells persist, enabling a rapid response upon re-exposure to the virus (21). Importantly, in some studies, vaccinated individuals often display broader and more potent antibody response compared to those with natural infections, underscoring the importance of vaccination in enhancing long-term immunity (22). High virus-specific antibody titers are associated with increased *in vitro* neutralization capacity and inversely correlated with viral load in patients (23). Nonetheless, high antibody levels have also been associated with increased disease severity, suggesting that a robust antibody response alone may not be sufficient to prevent severe clinical outcomes (24). Given that antibodies are not the primary defense mechanism against the virus, it is crucial to identify which immune components serve as reliable predictors of effective protection.

While mRNA vaccines initially confer high levels of protection, vaccine-induced immunity diminishes over time, a process further accelerated by the emergence of viral variants, leading to breakthrough infections. This underscores the importance of understanding long-term immune protection beyond humoral responses, with particular emphasis on the roles of T-cell-mediated immunity and epigenetic reprogramming of innate immune cells. Sustained and broad-spectrum protection likely depends on these mechanisms. Therefore, comprehensive profiling of T-cell responses and the identification of cellular correlates of protection remain key research priorities, especially as neutralizing antibody titers wane with time and ongoing viral evolution.

However, patients with severe or critical COVID-19 demonstrate 2.1- and 2.2-fold reduced absolute counts of CD4^+^ and CD8^+^ T cells, respectively, compared to those with moderate disease (25). The decrease in peripheral T cells is especially pronounced in the CD8+ T-cell population, but it remains uncertain whether this is due to these cells migrating to tissues with active SARS-CoV-2 replication, an increased rate of cell elimination, or inherently low baseline levels of these cells in individuals with severe disease (26). In addition to the quantitative reduction, patients with severe and critical COVID-19 often experience qualitative alterations in the T-cell compartment. The immune landscape in patients with fatal disease includes a marked contraction in lung effector and memory T cells, replaced by naïve T cells that are not fully equipped to counter the pathogens (27), suggesting a critical reset of the lymphoid compartment. This robust immune alteration is associated with high amounts of inflammation inducers (11, 27–30), and the presence of circulating immunoregulatory myeloid cells (27, 31–35), which together are the hallmarks of the severe stage of COVID-19. Building on this observation, promoting a robust vaccine-induced T-cell response is crucial for effective prevention.

mRNA vaccines stimulate robust activation of CD4^+^ T helper and CD8^+^ cytotoxic T lymphocytes (36–38). Interestingly, vaccination promotes the differentiation of both helper and cytotoxic T lymphocytes expressing a central memory 1 (TCM1)-like phenotype characterized as CCR7^+^CD27^+^CD45RA^-^ T cells (39). These cells can recirculate in peripheral tissues and be potentially more responsive after antigen re-encounter (39), but also persist for months to years, ensuring a swift immune response upon re-exposure to the virus (37). Moreover, vaccine-induced T cells demonstrate the ability to recognize various SARS-CoV-2 variants, offering cross-protection against emerging variants (37). Indeed, beyond spike-specific responses, a hybrid immunity results in the recognition of a much broader set of epitopes, including many non-spike antigens, which is also reflected in different T cell receptor (TCR) repertoires (40). Collectively, all these results suggest that the T-cell response plays a valuable role in reducing disease severity and controlling infection.

Although vaccines induce T-cell responses, our understanding of the specific T-cell phenotypes and functions that provide effective protection in real-world settings remains incomplete. In particular, the longitudinal behavior of these immune responses following multiple vaccine doses has yet to be fully characterized in large populations. This study, therefore, aimed to identify cellular biomarkers that predict immune protection in vaccinated healthcare workers. This analysis was conducted in a large cohort of healthcare workers (HW) across 4 European centers.

## Methods

### Population and available data

Five hundred and forty-six HW belonging to the University Hospitals of Padua, Perugia, Trieste, and Verona, and to the Public Health Institutes and Faculty Hospital in Banská Bystrica in Slovakia were enrolled in the study, which was carried out within the Horizon 2020 ORCHESTRA research project (41). The study was conducted between November 2020 and September 2022. Samples were collected at two time points: six months after the second dose (in the Perugia, Verona, and Trieste cohorts) or one month after the third dose (in the Padua and Slovakia cohorts). Samples that did not reach a lymphocyte (CD3^+^) cell count of 50,000 were excluded from the analysis. All subjects were vaccinated with at least two doses of a SARS-CoV-2 vaccine. Data on sociodemographic and clinical information were obtained through clinical records or an *ad-hoc* questionnaire. For each participant, the following information was collected: sex, age, job title, type and date of vaccination, date of infection, and comorbidities.

### SARS-CoV-2 specific T-cell responses

Peripheral blood mononuclear cells (PBMCs) were isolated from 9 mL heparinized blood with ORCHESTRA harmonized protocols using Cell Preparation Tubes (CPTs) and frozen at -80°C in foetal bovine serum (FBS)/10% dimethyl sulfoxide (DMSO). Samples were transported on dry ice with an in-transport temperature monitoring system for analyses at the University of Antwerp. On the day of analysis, PBMCs were thawed and rested overnight in RPMI 1640 medium (Gibco, Thermofisher Scientific, MA, USA) supplemented with 5% heat-inactivated AB serum (Sigma Aldrich), 100 U/mL penicillin (Biochrom, Berlin, Germany), and 0.1 mg/mL streptomycin (Biochrom). The cells were counted, and an activation-induced surface and cytokine marker assay was performed as described for other studies within ORCHESTRA (42, 43). For stimulation, a pool of lyophilized peptides was utilized. The pool was of 15-mer sequences with 11 amino acids overlapping, covering the immunodominant sequence domains of the spike glycoprotein (“Protein S”; Prot_S, GenBank MN908947.3, Protein QHD43416.1) of SARS-CoV-2 (Miltenyi Biotech, Leiden, Netherlands). The peptide pool was utilized at 1 μg/mL. Unstimulated negative controls were performed with equal volumes of sterile water and 10% DMSO. This was added at a 1:100 dilution to the culture wells, resulting in a final DMSO concentration of 0.05%, to normalize positively stimulated cells. Cell activation cocktail, composed of PMA (phorbol-12-myristate 13-acetate) and ionomycin (Bio Legend, Amsterdam, Netherlands) in DMSO, was used as a positive control. Incubation of PMBCs was performed at 37 °C, 5% CO_2_ for 2 hours after which 2 μg/mL brefeldin A (BioLegend, Amsterdam, Netherlands) was added and further incubation for 4 hours at 37 °C and 5% CO_2_ before being collected for flow cytometric analysis. Thereafter, cells were stained with Zombie aqua fixable viability dye (BioLegend) for 15 minutes in the dark at room temperature and washed with cell staining buffer (PBS, 1% bovine serum albumin, 2 mM EDTA).

### T-cell staining and flow cytometry

Cells were stained with surface antibody mixture including anti-CD3-APC Fire750, anti-CD4-FITC, anti-CD8a-BV570, anti-CD154-APC, and anti-CD45RO-PerCP/Cyanine5.5 (BioLegend) for 15 minutes at room temperature (**Supplementary Table S1**). Afterwards, cells were washed with cell staining buffer and fixed/permeabilized using inside stain kit following manufacturer’s instructions (Inside stain kit, Miltenyi Biotec) for 20 minutes in the dark at room temperature. Cells were then further washed with permeabilization buffer and stained with antibodies directed towards intracellular cytokines (anti-IFNγ-PE, anti-TNFα-PE/Cyanine7 and anti-IL-2-BV421, BioLegend) for 15 minutes in the dark at room temperature. Finally, cells were washed in cell staining buffer. Flow cytometry (FC) was performed on NovoCyte Quanteon 4025 flow cytometer (Agilent, CA, USA), and FC data were analyzed using FlowJo v10.8.1 (BD Biosciences, CA, USA). Gating strategy was performed on an auto-generated ancestry algorithm available in FlowJo v10. Fluorescence compensation was performed using the NovoExpress v1.6.1 software integrated with the NovoCyte Quanteon. Single-stained controls were acquired under identical instrument settings, and NovoExpress automatically computed and applied a compensation matrix based on median fluorescence intensity values for each fluorochrome. The matrix was visually inspected and, if required, minimally adjusted to ensure optimal separation before export to FlowJo for analysis.

Gating strategy included the following sequential steps: (i) initial gating on time versus scatter to exclude acquisition artifacts, (ii) doublet discrimination based on forward scatter area (FSC-A) versus height (FSC-H) to include only singlets, and (iii) exclusion of dead cells based on Zombie Aqua viability staining. Live, single CD3^+^ T cells were then subdivided into CD4^+^ and CD8^+^ subsets for downstream analysis of activation markers (CD154, CD45RO) and intracellular cytokine expression (IFNγ, TNFα, IL-2) (**Supplementary Figure S1**) (42, 43). To account for inter-sample variability in basal activation, cytokine expression was quantified as the net response, calculated by subtracting the cytokine level in unstimulated (steady-state) samples from that in samples stimulated with the specific spike-specific peptides pool. This approach minimizes the influence of background cytokine release and allows accurate evaluation of peptide-induced activation using flow cytometry.

Fluorescence Minus One (FMO) controls were included for all cytokine and activation markers to define gating boundaries for positive populations. A “self-generated ancestry algorithm” in FlowJo was used to visualize the hierarchical gating workflow constructed based on our FMO-defined gating sequences. In this analysis, the ancestry tree was generated by FlowJo to maintain consistent hierarchical relationships among gates (e.g., Live → Singlets → CD3^+^ → CD4^+^/CD8^+^ → cytokine^+^ subsets), ensuring reproducibility and traceability across samples. For cellular immunity analyses, a minimum of 50,000 CD3^+^ cells per sample was required as a quality control (QC) threshold.

### Statistical methods

Quantitative variables were summarized as medians and interquartile ranges (IQR) due to non-normal distribution, and categorical variables as percentages. Spearman’s correlation was used to assess associations between quantitative variables, while the Kruskal-Wallis test was performed to evaluate differences between categorical variables and the median of quantitative variables.

Cox proportional hazards models were used to assess the association between post-infection status (outcome) and immune cell levels, adjusting for sex, age, and history of previous infections in Verona and Perugia centers. Cells were categorized into tertiles with the 2nd tertile serving as the reference group. Separate Cox models were performed, one including all subjects, and another excluding individuals with infections both before and after vaccination. Statistical analyses were performed using STATA^®^ version 18.0 (StataCorp, College Station, Texas, USA).

## Results

### Study population

A total of 546 HW samples were analyzed, of which 305 (56%) reached the inclusion criteria of 50,000 CD3+ cells (mean T-cell viability = 68%), and were included in the final analysis (Padua 11/100, Perugia 16/32, Slovakia 52/74, Trieste 0/39, Verona 226/301) (**Supplementary Figure S2**). Most participants (74%) were recruited from the Verona cohort, reflecting a real-world, region-specific setting that, while valuable, may not fully capture broader population heterogeneity. Of the studied population, approximately 69.8% were female, and 27.2% had a SARS-CoV-2 infection either before or after sampling (14.4% and 12.8%, respectively). The median age of the participants was 47.2 years (p25-p75 = 29.6-56.9). The most prevalent job category was “Other HW”, which included all health professionals not classified in other categories, such as psychologists, physiotherapists, pharmacists, midwives, public health specialists, hygienists, epidemiologists, etc. This was followed by categories of nurses and administrative staff. All sociodemographic characteristics and infectious statuses of participants are detailed in **Table 1**. More than 90% of HW were vaccinated with BNT162b2 (Comirnaty). Only three subjects completed the primary course with a dose of Jcovden (Janssen). Data on vaccine type is not available for 10 subjects.

**Table 1:** Sociodemographic characteristics and infectious status of the health workers included in the analysis.

	N.	%
Cohort		
Verona	226	74.1
Slovakia	52	17.1
Perugia	16	5.3
Padua	11	3.6
Age – median (p25, p75)	47.2	(29.6; 56.9)
Sex		
Female	213	69.8
Male	92	30.2
Job title		
Administrative	45	14.8
Nurse	63	20.7
Other HW	127	41.6
Physician	37	12.1
Technician	33	10.8
Infection		
Never infected	222	72.8
Infected before sampling	44	14.4
Infected after sampling	39	12.8

**Figure 1:**
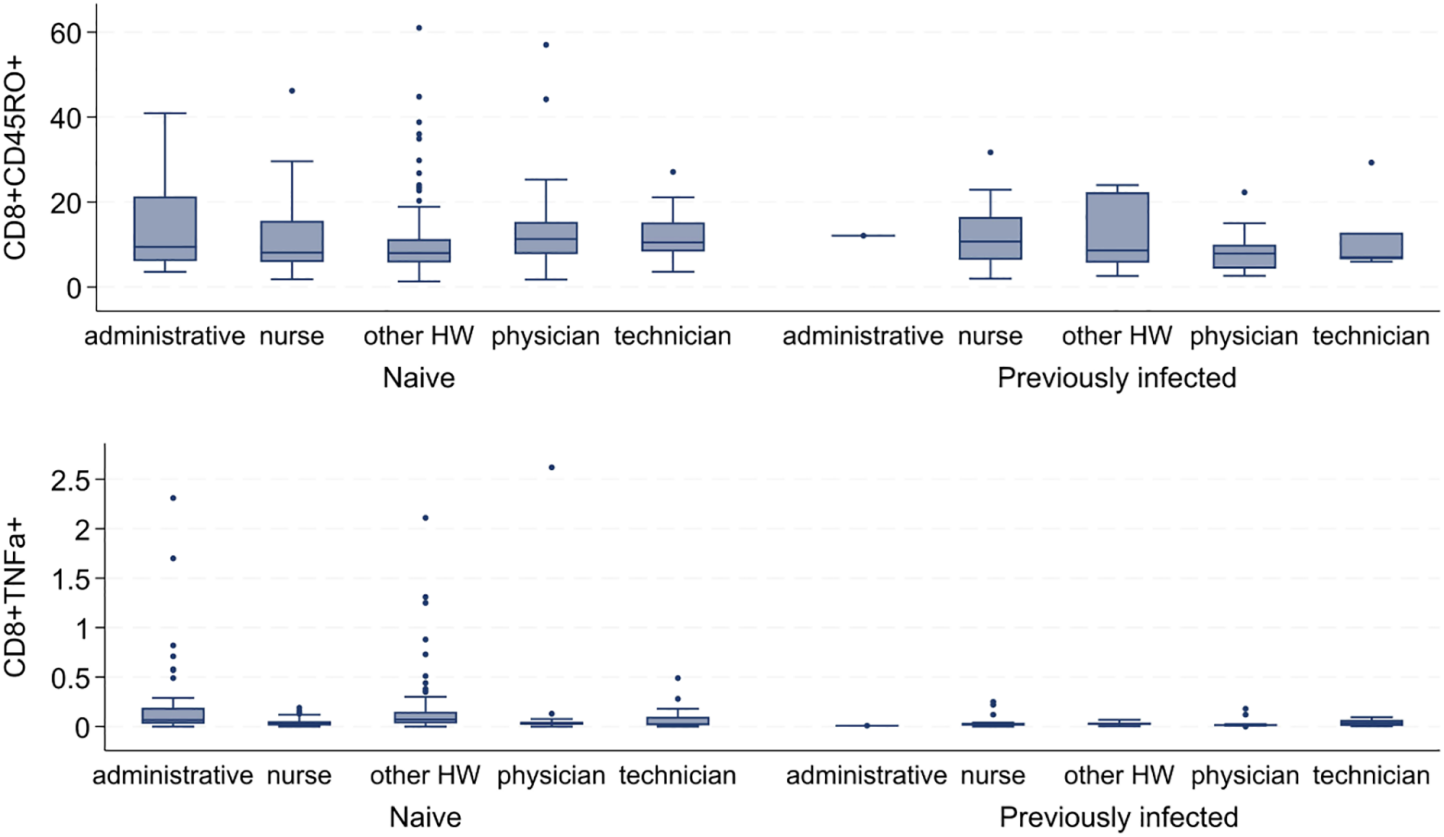
CD8^+^CD45RO^+^ and CD8+TNFα^+^ median levels of expression in response to SARS-CoV-2 spike peptides by category in naïve and previously infected HW.

Using intracellular staining (ICS) protocol on conventional flow cytometry, we measured the frequency of Interferon (IFN)-γ, Tumor Necrosis Factor (TNF)-α, and Interleukin (IL)-2-producing T lymphocytes in two main subsets of T lymphocytes: CD4^+^ and CD8^+^ T cells in response to spike and nucleocapsid SARS-CoV-2 peptides. Moreover, we defined the functional activation of T cells by exploiting the expression of the CD40L (CD154) marker, whereas we used the expression of CD45RO to define T cell differentiation in the periphery. This report highlighted the frequency of effector spike-specific T lymphocytes defined as CD40L T cells and memory T lymphocytes identified as CD4^+^CD45RO^+^ or CD8^+^CD45RO^+^ cells. Considering the age impact on the modulation of the immune response after vaccination, we found that only the cell population of spike-specific cytotoxic CD8^+^ T cells characterized as CD8^+^CD45RO^+^ and CD8^+^TNFα^+^ T cells in subjects naïve (not previously infected) had a significant correlation with increasing age. Specifically, the frequency of spike-specific CD8+CD45RO+ T lymphocytes showed a weak positive correlation with age (Spearman’s rho = 0.34, p<0.001), whereas the frequency of spike-specific CD8+TNFα+ T cells was negatively correlated with age (Spearman’s rho = -0.23, p<0.01). Finally, we considered possible influences on the vaccination-dependent immune response based on participants’ job categories. As previously reported, the only cell subsets that showed a significant trend were the CD8^+^CD45RO^+^ and CD8^+^TNFα^+^ T cells in naϊve HW. Indeed, the median expression of CD8^+^CD45RO^+^ T cell ranged from 7.99 in the other HW category to 11.3 in the physician category (p=0.02). On the contrary, the median level of CD8^+^TNFα^+^ T cell was lowest in the technician category (Spearman’s rho =0.024) and the highest level in the other HW job category (Spearman’s rho =0.072) (p<0.001) (**Figure 1**). In our analysis of CD4^+^ T cell subsets, we found that CD4^+^CD45RO^+^ cells showed a positive correlation with age in previously uninfected HW (Spearman’s Rho = 0.38; p < 0.001). Conversely, CD4^+^IFNγ^+^ cells exhibited a negative correlation with age in this same group (Spearman’s Rho = -0.32; p < 0.001). Additionally, we did not observe significant correlations between sex and the cytokine release from either CD4^+^ or CD8^+^ T cells. Overall, our findings indicate that all vaccinated individuals, regardless of their job category, did not show significant differences in the anti-spike cytotoxic cellular immune response.

### Anti-spike-specific, cellular immunity features in vaccinated HWs

We profiled the circulating immune response, with particular emphasis on identifying potential perturbation of the cytotoxic T-cell compartment. First, we assessed possible correlations between cytokine-producing, spike-specific T cell subsets and the functional/differentiation status of these antigen-specific T cells. As shown in **Table 2**, most correlations were non-significant and ranged from moderate to low. In contrast, a moderate but significant correlation was found between both memory T cell subsets (CD4^+^CD45RO^+^ vs CD8^+^CD45RO^+^ cells: r = 0.659, p<0.01) and IL2-producing T cells (CD4^+^IL-2^+^ vs CD8^+^IL-2^+^ cells: r = 0.600, p<0.01). Collectively, this analysis did not display correlation in cytokine-producing cells between the CD4^+^ and CD8^+^ T cells subsets following vaccination (**Table 2**).

**Table 2:** Spearman’s correlation analysis among cytokines-expressing and memory phenotype of CD8^+^ and CD4^+^ T cells, age, and days in naïve health workers.

	CD4+ CD45RO	CD4+ CD154+	CD4+ IL2+	CD4+ IFNy+	CD4+ TNFa+	CD8+ CD45RO	CD8+ CD154+	CD8+ IL2+	CD8+ IFNy+	CD8+ TNFa+	Age (ys)	Lag-time between last dose and sampling (dd)
CD4+CD45RO+	1											
CD4+CD154+	0.0399	1										
CD4+IL2+	0.0047	0.1341	1									
CD4+IFNy+	-0.0535	0.1531	0.4443*	1								
CD4+TNFa+	0.1979*	0.2519*	0.3309*	0.2465*	1							
CD8+CD45RO	0.6604*	0.0661	0.0133	-0.0382	0.121	1						
CD8+CD154+	0.0495	0.6622*	-0.072	-0.0127	0.1098	0.0353	1					
CD8+IL2+	-0.022	-0.0818	0.5998*	0.1760*	0.0216	-0.0105	-0.1466	1				
CD8+IFNy+	-0.0293	0.1960*	0.0955	0.2921*	0.0168	-0.0431	0.1815*	-0.004	1			
CD8+TNFa+	-0.0218	0.2785*	0.3056*	0.3362*	0.2654*	-0.0992	0.2129*	0.1863*	0.2038*	1		
Age (ys)	0.3764*	-0.1061	-0.008	-0.3169*	-0.0769	0.3415*	-0.0411	0.0222	-0.0625	-0.2339*	1	
Lag-time between last dose and sampling (dd)	0.0338	0.0517	-0.1684*	-0.2351*	-0.0785	0.0078	0.1326	-0.1622	0.0663	-0.1942*	0.2960*	1

*p < 0.01.

**Figure 2:**
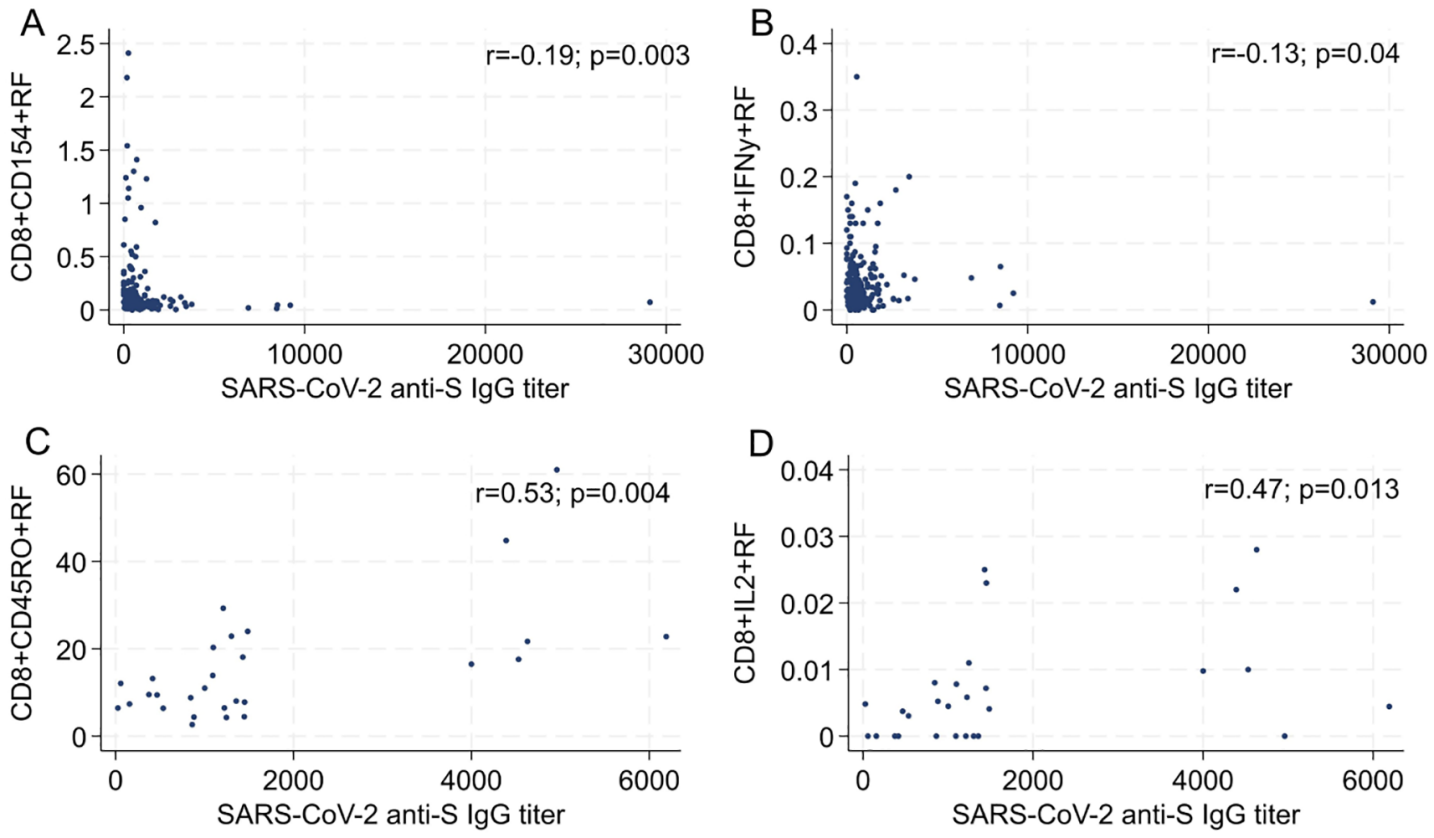
Correlation between percentage of CD8 T cell response (cytokines and functional stage) and anti-S SARS-CoV-2 IgG titer in Verona and Perugia **(A, B)**, Padua and Slovakia **(C, D)** cohorts.

**Figure 3:**
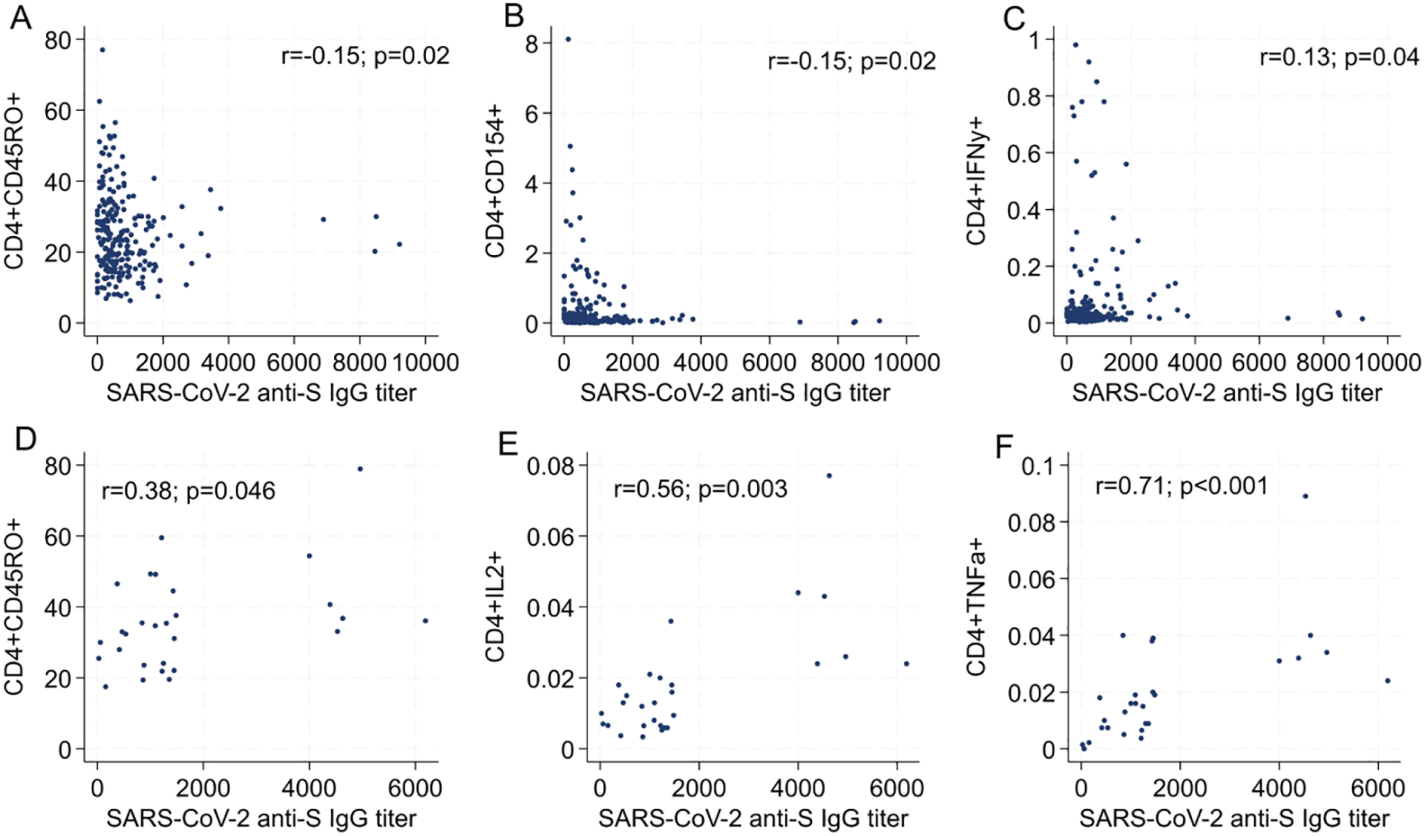
Correlation between percentage of CD4 T cell response (cytokines and functional stage) and anti-S SARS-CoV-2 IgG titer in Verona and Perugia cohorts **(A–C)**, and Padua and Slovakia cohorts **(D–F)**.

Next, we examined the correlation between cytokine-producing, spike-specific T cell subsets and the differentiation stage in relation to the anti-spike humoral response (**Figure 2**). A distinct pattern emerged across the different cohorts. In the Verona and Perugia cohorts (**Figures 2A, B**), we observed a negative correlation between the serum anti-spike IgG titers and the frequency of both effector CD8^+^ T lymphocytes (CD8^+^CD154^+^ cells) and IFN-γ-producing CD8^+^ T lymphocytes (CD8^+^IFNγ^+^ cells) (**Supplementary Figure S3**). In contrast, a positive correlation was found between the serum anti-spike IgG titers and the aforementioned T cell subsets in the Padua and Slovakia (**Figures 2C, D**) cohorts. The discrepancy between cohorts likely stems from differences in sampling timing (6 months post-second dose vs. 1 month post-third dose), underscoring the temporal dynamics of T-cell responses following vaccination. In the same way, the correlation between CD4^+^ lymphocytes and anti-spike humoral response depended on the sampling time point. Indeed, CD4^+^CD45RO^+^ and CD8^+^CD154^+^ cells negatively correlated with antibody titer in the Verona and Perugia cohorts (Spearman’s rho = -0.15), even though the correlation was very weak (**Figures 3A, B**). Similarly, a weak or moderate positive correlation was shown between CD4^+^CD45RO^+^ T cells, CD4^+^IL2^+^T cells, and CD4^+^TNFα^+^ T and the humoral response in the Slovakia and Padua cohorts (Spearman’s rho = 0.38, 0.56, and 0.71, respectively) (**Figures 3D–F**). The only exception was the CD4^+^IFNγ^+^ T cell subset, which had a positive correlation at the first time point (**Figure 3C**). Notably, this divergent pattern corresponds to the different time points of analysis post-vaccination: participants from the Verona and Perugia cohorts were analyzed 6 months after the second vaccine dose, whereas those from Padua and Slovakia were assessed 1 month after their third vaccine dose.

**Figure 4:**
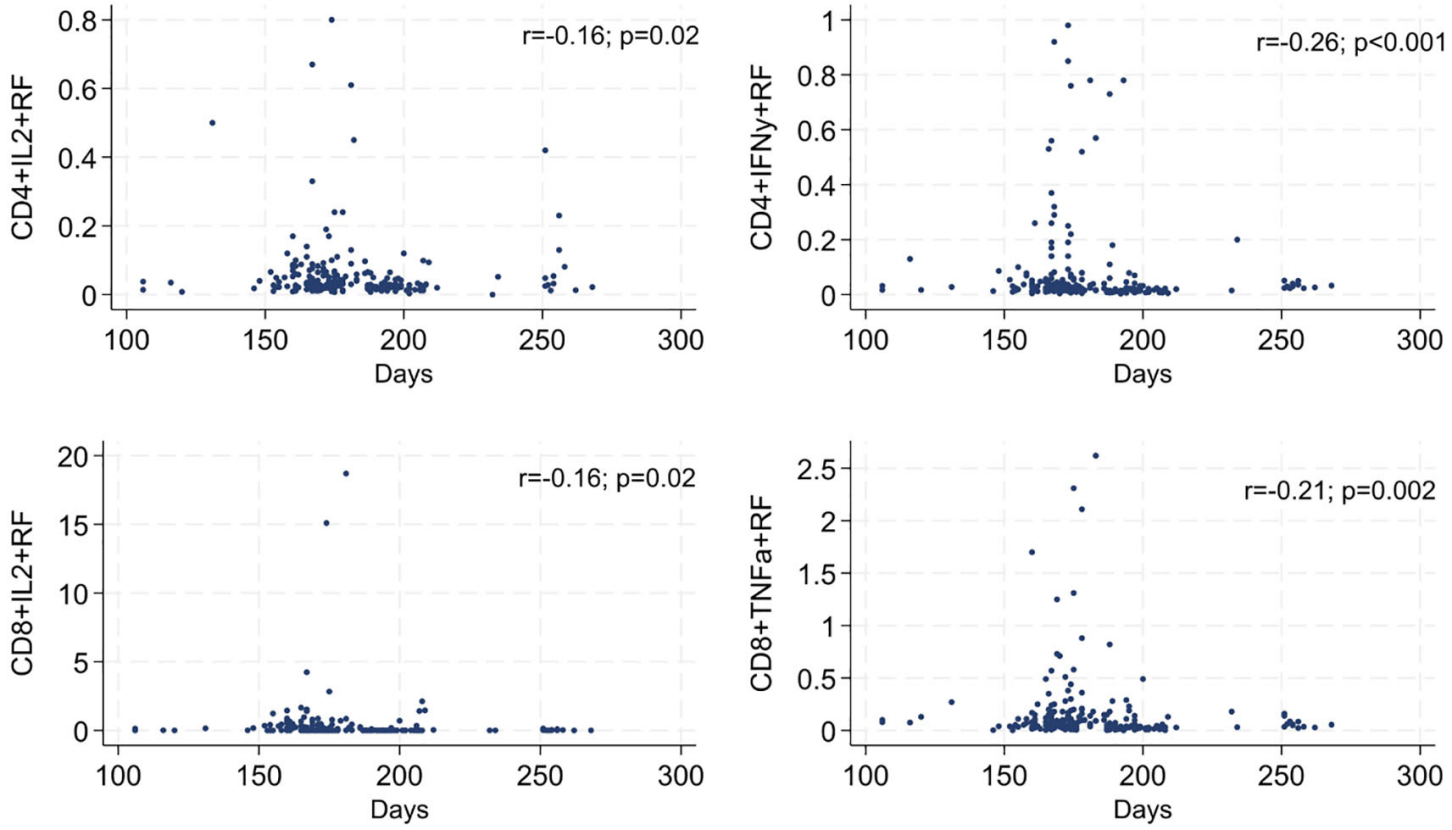
Significant correlation between T cell-dependent cytokines and time elapsed since the 2^nd^ dose.

Next, the study investigated whether the time elapsed between the administration of the last vaccine dose and sample collection influenced cytokine release by CD8^+^ and CD4^+^ T cells. This analysis was performed exclusively in the Perugia and Verona cohorts, which had a broader temporal range of sampling. A significant negative correlation with time was observed only for IL-2- and TNF-α-producing CD8^+^ T cells, and IL-2- and IFNγ-producing CD4^+^ T cells, with Spearman’s rho of -0.1573, -0.2123, -0.16, and -0.26, respectively (**Figure 4**). This trend is consistent with patterns observed in vaccine-induced immunity, where, after the initial immune activation, the immune system enters a phase of immune memory, where T cells are maintained in a less activated state; however, these cells can respond quickly if re-exposed to the pathogen. Thus, the observed decline in cytokine production likely reflects a shift from active defense to long-term immune surveillance—a key feature of effective immunity. However, the gradual nature of this decline highlights the durability of immune memory, even as the immediate cytokine responses wane.

Building on these findings, the analysis was extended to assess the presence of cytokine-producing and effector/memory CD8^+^ T cells across three groups defined by infection status: never infected, infected prior to sampling, and infected after sampling.

**Figure 5:**
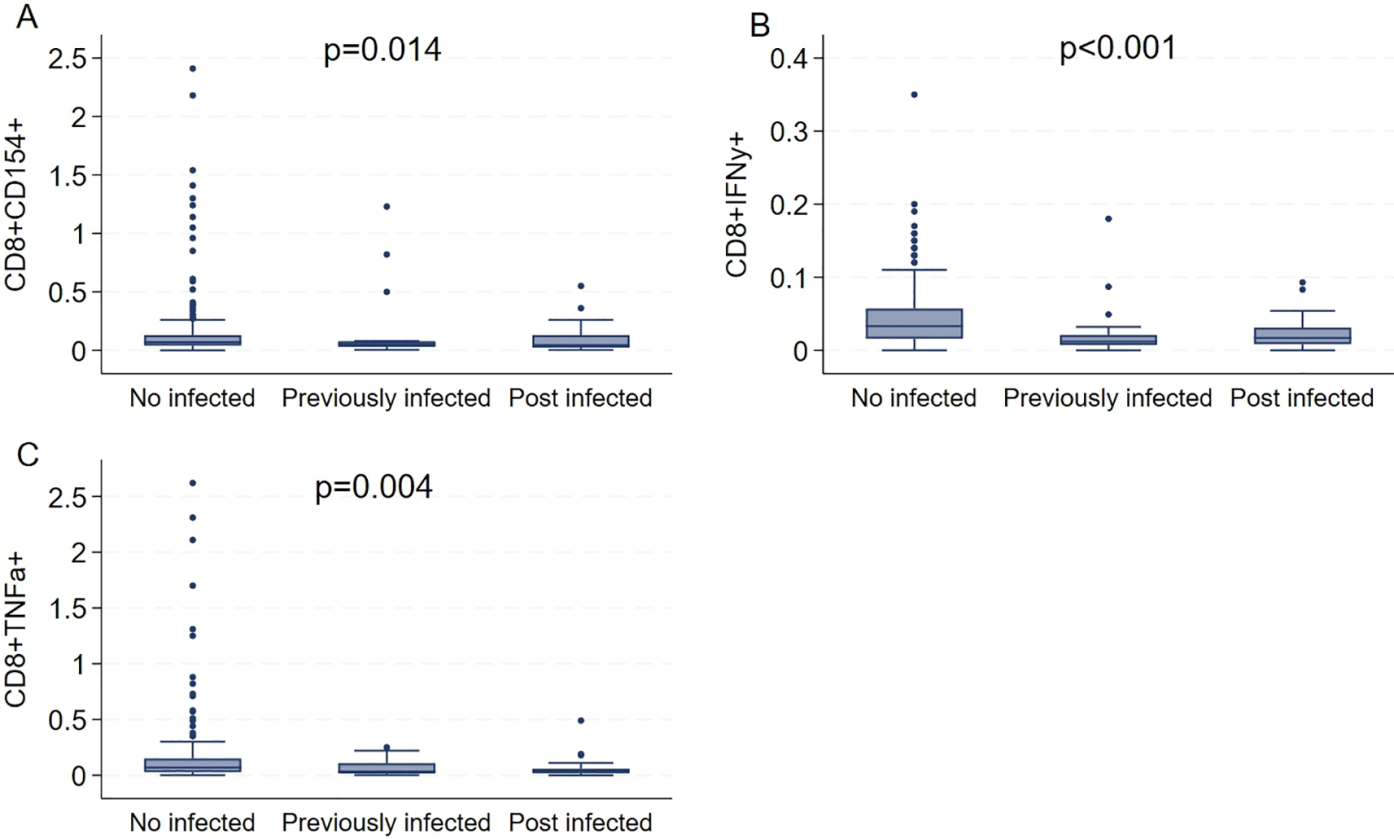
Median levels of cytokine-associated and memory phenotype of CD8^+^ T cells in health workers 6 months after the 2^nd^ dose **(A–C)**, by infectious status.

By clustering the cytotoxic-associated immunity, in the cohorts tested 6 months after the second dose, the frequency of activated (CD8^+^CD154^+^, **Figure 5A**), cytokine-producing (CD8^+^IFNy^+^, **Figure 5B**), memory (CD8^+^CD45RO^+^, **Figure 5C**) T cell subsets was significantly higher in naïve subjects, compared to the other categories. The unexpected nature of this result could reflect a combination of factors, including the timing of sample collection, distinct immune imprinting patterns, and technical variables such as cryopreservation-induced cell loss. Collectively, these data suggest that vaccination can induce a specific immune response against the antigen. This primary response (after the first exposure to the vaccine) appears to last longer than the secondary immune response (which happens when the immune system encounters the same antigen again), as observed in individuals infected either before or after vaccination.

Finally, a Cox regression model was performed to assess associations between the potential risk of infection and T-cell immunity (**Table 3**). Interestingly, the only CD8^+^ T cell subset whose median level correlated with the risk of infection was the activated/effector T cell population, identified as CD8^+^CD154^+^ lymphocytes. Indeed, HWs with median levels of CD8^+^CD154^+^ lymphocytes in the 1^st^ tertile had a Hazard Ratio (HR) of 2.64.(C.I. 95% 1.05-6.65), compared to those in the 2^nd^ tertile. This suggests that individuals who did not mount an effective response after vaccination were more susceptible to infection. Another non-immune parameter that correlated with the risk of infection was age, where older subjects showed a higher likelihood of infection.

**Table 3:** Cox models for the risk of infection after the sampling for CD8+CD154+ median levels, expressed as tertile categories (tertiles were calculated only for the Verona and Perugia centers; tertile 2 was used as reference).

	Tertile	Cells range	All subjects (n=242)				Group A* (n=237)			
			N. of infections	HR	IC95%	P	N. of infections	HR	IC95%	P
CD4+CD45RO+	1	6.27-18.2	7	0.46	0.18-1.17	0.104	7	0.52	0.20-1.34	0.177
	2 (ref.)	18.4-28.3	13	1			12	1		
	3	28.6-77	12	0.68	0.31-1.52	0.347	8	0.48	0.19-1.18	0.109
	Age			1.03	1.00-1.06	0.033		1.04	1.01-1.07	0.016
CD4+CD154+	1	0-0.056	15	1.54	0.61-3.88	0.360	14	1.96	0.69-5.56	0.204
	2 (ref.)	0.057-0.18	7	1			5	1		
	3	0.19-8.11	10	1.71	0.64-4.52	0.282	8	1.85	0.60-5.67	0.281
	Age			1.03	1.00-1.06	0.034		1.03	1.00-1.07	0.032
CD4+IL2+	1	0-0.022	10	0.53	0.22-1.26	0.150	8	0.58	0.23-1.47	0.246
	2 (ref.)	0.023-0.043	12	1			10	1		
	3	0.044-0.8	10	0.78	0.33-1.84	0.572	9	0.85	0.34-2.12	0.722
	Age			1.03	1.00-1.06	0.025		1.04	1.01-1.07	0.020
CD4+IFNy+	1	0.003-0.017	18	1.57	0.69-3.57	0.286	16	1.93	0.78-4.76	0.153
	2 (ref.)	0.018-0.034	9	1			7	1		
	3	0.035-0.98	5	0.80	0.26-2.45	0.700	4	0.86	0.25-3.00	0.810
	Age			1.03	1.00-1.06	0.046		1.03	1.00-1.07	0.036
CD4+TNFa+	1	0.004-0.021	13	1.31	0.55-3.12	0.543	11	1.36	0.55-3.41	0.506
	2 (ref.)	0.022-0.038	9	1			8	1		
	3	0.0.39-0.37	10	1.37	0.55-3.40	0.496	8	1.20	0.45-3.22	0.711
	Age			1.03	1.00-1.06	0.027		1.04	1.01-1.07	0.021
CD8+CD45RO	1	1.31-6.43	12	1.75	0.69-4.47	0.239	12	2.12	0.80-5.66	1.32
	2 (ref.)	6.51-10.5	7	1			6	1		
	3	10.7-44.2	13	1.57	0.62-3.95	0.340	9	1.26	0.45-3.58	0.658
	Age			1.03	1.00-1.06	0.032		1.04	1.01-1.07	0.016
CD8+CD154+	1	0-0.047	19	2.64	1.05-6.65	0.040	16	3.40	1.13-10.21	0.030
	2 (ref.)	0.048-0.096	6	1			4	1		
	3	0.097-2.41	7	1.40	0.47-4.21	0.545	7	2.03	0.59-6.95	0.261
	Age			1.03	1.00-1.06	0.042		1.03	1.00-1.07	0.029
CD8+IL2+	1	0-0.005	13	1.53	0.65-3.60	0.332	10	1.39	0.55-3.54	0.487
	2 (ref.)	0.005-0.028	9	1			8	1		
	3	0.031-18.7	10	1.34	0.53-3.38	0.541	9	1.34	0.50-3.59	0.555
	Age			1.03	1.00-1.06	0.027		1.04	1.01-1.07	0.021
CD8+IFNy+	1	0-0.017	17	1.52	0.63-3.66	0.353	14	1.37	0.57-3.30	0.482
	2 (ref.)	0.018-0.042	8	1			8	1		
	3	0.043-0.35	7	0.93	0.34-2.57	0.890	5	0.70	0.23-2.14	0.529
	Age			1.03	1.00-1.06	0.032		1.04	1.00-1.07	0.024
CD8+TNFa+	1	0-0.036	17	1.34	0.59-3.07	0.484	14	1.15	0.47-2.83	0.753
	2 (ref.)	0.037-0.095	9	1			8	1		
	3	0.098-2.62	6	0.67	0.24-1.89	0.449	5	0.60	0.20-1.85	0.378
	Age			1.03	1.00-1.06	0.035		1.0.4	1.00-1.07	0.025

*Subjects infected before and after the sampling were excluded (n=5).

## Discussion

The T cell response to SARS-CoV-2 infection plays a crucial role in immune defense, aiding both viral clearance and long-term immunity. Indeed, the cellular immune system has specifically evolved to identify and control intracellular pathogens. CD8^+^ T cells are responsible for eliminating infected cells, while CD4^+^ T cells help coordinate and enhance the overall immune reaction, as well as support the generation and maintenance of high-affinity anti-SARS-CoV-2 IgG antibodies (44). For this reason, the effectiveness of anti-SARS-CoV-2 vaccination relies on the induction of a T cell response (45). Interestingly, protective clinical effects are observed within 11 days after the first vaccination, with a strong CD8^+^ T cell response detectable during this early period. This suggests that the CD8^+^ T cell response may play a key role in, or at least contribute to, these early observations of protection (38). Therefore, characterizing the T-cell response should be a fundamental aspect of vaccine development. At present, evaluating T cell-specific immune responses in vaccinated individuals is limited by the high cost and technical demands of assays such as flow cytometry and ELISPOT, which are not easily integrated into standard diagnostic workflows. These methods require fresh or cryopreserved PBMCs, thus involving more complex infrastructure for sample handling and storage compared to the simpler requirements for assessing circulating antibodies or soluble factors from serum or plasma. Consequently, such analyses are primarily confined to translational research rather than routine clinical diagnostics.

This study showed that the anti-spike cellular response is dynamic and can be influenced by various factors, including the time elapsed since the vaccination boost and the number of doses received. One interesting finding from this report is that the number of CD8^+^CD154^+^ T cells, which resemble activated T cells, can predict the risk of infection. CD40L, also known as CD154, is traditionally expressed on CD4^+^ T cells and serves as a key modulator of both humoral and cellular immune responses (46). However, studies involving CD40L-deficient (CD40L^−/−^) mice have shown that the absence of CD40L leads to reduced numbers of memory CD8^+^ T cells, suggesting the crucial role of CD40L in the generation of protective memory CD8^+^ T cells (47). In particular, CD40L upregulation allows CD8^+^ T cells to promote their own expansion and differentiation in a cell-extrinsic manner (48). The acquisition of CD154/CD40L is a crucial step in the differentiation of T cells from naïve to effector/memory T cells (46). Therefore, both the absence and the reduction of effector/memory T cells have been reported as a characteristic feature of COVID-19 and may serve as a predictor of disease severity (12, 27, 49, 50). In general, patients with severe or critical COVID-19 exhibit a robust reduction in the absolute numbers of CD4^+^ and CD8^+^ T cells, respectively, compared to those with moderate disease. This decrease in peripheral T cells, especially within the CD8^+^ T cell compartment, is particularly pronounced (27, 51). However, the cause of this reduction remains uncertain, with possible explanations including the trafficking of these cells to tissues with ongoing SARS-CoV-2 replication, increased elimination of the cells, or pre-existing low levels of T cells in individuals who develop severe disease.

Interestingly, we observed that naïve vaccinated individuals exhibited a higher frequency of activated/memory CD8^+^ T cells compared to those with hybrid immunity, a finding that contrasts with most reports suggesting enhanced T-cell responses in individuals with prior SARS-CoV-2 infection (37). While unexpected, this discrepancy may be attributed to several factors. First, the timing of sample collection differed across subgroups and may have captured distinct phases of the T-cell response: for example, contraction of the effector pool may have already occurred in hybrid individuals sampled later post-infection or post-vaccination. Second, differences in immune imprinting and antigenic exposure. Indeed, repeated stimulation in hybrid immunity, potentially promoting T-cell exhaustion or altered differentiation states, could influence memory phenotypes. Third, methodological factors, including the markers used to define activation/memory subsets, batch effects, or cryopreservation-related cell loss, may have contributed to this observation. While the current dataset does not allow definitive conclusions, these findings highlight the complexity and heterogeneity of vaccine-induced cellular immunity and underscore the need for further stratified analyses in larger, well-characterized cohorts. To definitively determine if CD8^+^CD154^+^ cells constitute a distinct, stable subset with helper-like functions or simply reflect a temporary activation state within the overall CD8^+^ T cell pool, further detailed studies are necessary.

We did not observe the impact of gender on specific T-cell response at any time point or any of the analyzed cell subsets, aligning our findings with other published reports (52–54). On the other hand, we detected age-related changes in both CD4^+^ and CD8^+^ subsets. These findings align with the concept of immunosenescence, whereby aging reduces the functional capacity of effector T cells while driving expansion of memory compartments. Moreover, we did not observe any correlation in cytokine-producing cells between the CD4^+^ and CD8+ T cell subsets. We noted only that the ability of CD8^+^ T cells to produce inflammatory cytokines diminishes over time following vaccine administration, which is in line with previous reports (55). We identified a time-dependent divergent correlation between the anti-spike humoral response and the activation state of CD8^+^T cells, measured by CD8^+^CD154^+^ or CD8^+^IFNγ^+^ cells. Specifically, a positive correlation was present in the cohort tested 6 months after the last vaccination, whereas a negative trend was observed in the cohort tested after 1 month post-vaccination. Finally, we confirm that the T-cell response is more durable than the antibody response, consistent with previous studies that describe cellular adaptive immunity as less susceptible to viral variants (56, 57).

This study has several strengths. The enrolled cohort is substantial in size and primarily composed of healthy HWs, minimizing confounding factors such as comorbidities or pharmacological treatments that could affect the immune response to vaccination. This well-defined and homogeneous group of vaccinated individuals was extensively phenotyped, providing a robust foundation for immune response analysis. Longitudinal data were collected from participants who received varying numbers of vaccine doses and were analyzed at different time points post-vaccination, enabling us to track the evolution of vaccine-induced immune response. Additionally, the large cohort size allows us to use statistical models to assess correlations between multiple immune markers and clinical features. However, this work has some limitations. First, the limited panel of phenotypic markers constrained our ability to thoroughly categorize T cell subsets. Second, we evaluated only a small number of cytokines relevant to Th polarization. Third, our analysis focused exclusively on the immune response to the spike antigen. Four, the majority of participants (74%) were enrolled from the Verona cohort, potentially limiting geographic diversity. Moreover, as with any observational study, we cannot rule out the possibility that the observed associations are influenced by residual confounding. Furthermore, the sampling timepoints varied among the Cohorts. While this allowed us to investigate two different periods of the pandemic (before and after the booster dose), on the other hand, it limits the comparability of the results. Finally, we did not evaluate the anti-N titer to detect previous infection. Indeed, past infections were investigated only by screening or post-exposure swab testing and by self-reported information. As a result, the number of previous infections, especially the asymptomatic ones, could have been under-estimated.

In conclusion, this study demonstrates that CD8-restricted immunity induced by vaccination follows a dynamic pattern, where CD154 expression—a key marker associated with T-cell activation after antigen stimulation—correlates with protection against infection. Individuals with lower levels of CD8^+^CD154^+^ T cells were at greater risk of infection, highlighting the importance of an effective effector response. Our findings indicate that future public health strategies for booster vaccination should incorporate assessments of T cell activation, with a particular emphasis on validating the predictive value of CD8^+^CD154^+^ T cells in larger, more diverse populations. A comprehensive functional characterization of this subset is also essential to identify individuals who are most likely to benefit from additional booster doses. However, further research is needed to explore the mechanisms that drive this dynamic T-cell response and to define the TCR-clonotypes expanded by vaccination.

## Data Availability

The datasets generated during the current study are not publicly available because they contain sensitive data to be treated under data protection laws and regulations. Appropriate forms of data sharing can be arranged after a reasonable request to the last author (Stefano Porru; mail address: stefano.porru@univr.it).

## WP5 Working Group

Maria Luisa Scapellato, Department of Cardio-Thoracic-Vascular Sciences and Public Health, University of Padova, 35128 Padova, Italy, and Unit of Occupational Health, University Hospital, 35128 Padova, Italy; Manuela Campisi, Department of Cardio-Thoracic-Vascular Sciences and Public Health, University of Padova, 35128 Padova, Italy; Luana Cannella, Department of Cardio-Thoracic-Vascular Sciences and Public Health, and Occupational Medicine Unit, University Hospital of Padova, 35128 Padova, Italy; Antonia Franceschi, Section of Occupational Medicine, Department of Diagnostics and Public Health, University of Verona, 37134 Verona, Italy; Roberto Cannalire, Section of Occupational Medicine, Department of Diagnostics and Public Health, University of Verona, 37134 Verona, Italy; Melissa Ferrian, Section of Occupational Medicine, Department of Diagnostics and Public Health, University of Verona, 37134 Verona, Italy; Petra Hellebrandt, Dpt. Of Epidemiology, RAPH, Banská Bystrica, Blanka Hovoričová; Ludek Hochmuth, Faculty Hospital in Banská Bystrica, Slovak Republic (FDRH in B. Bystrica).

## WP6 Working Group

Vincent Faes, Molecular Pathology Group, Cell Biology & Histology, Faculty of Medicine and Health Sciences and Laboratory of Medical Microbiology, Vaccine & Infectious Disease Institute, University of Antwerp, Antwerp, Belgium; Vincent Van averebeke, Molecular Pathology Group, Cell Biology & Histology, Faculty of Medicine and Health Sciences and Laboratory of Medical Microbiology, Vaccine & Infectious Disease Institute, University of Antwerp, Antwerp, Belgium; Matilda Berkel, Molecular Pathology Group, Cell Biology & Histology, Faculty of Medicine and Health Sciences and Laboratory of Medical Microbiology, Vaccine & Infectious Disease Institute, University of Antwerp, Antwerp, Belgium.

## Glossary

CPTs: Cell Preparation Tubes
PBS: cell staining buffer
TCM1: central memory 1
CTLs: Cytotoxic T cells
DMSO: dimethyl sulfoxide
FC: Flow cytometry
FMO: Fluorescence Minus One
FBS: Foetal Bovine Serum
FSC-A: Forward Scatter Area
FSC-H: Forward Scatter Height
HR: Hazard Ratio
HW: Healthcare Workers
IFN: Interferon
IL: Interleukin
IQR: Interquartile Ranges
ICS: Intracellular Staining
PBMCs: Peripheral blood mononuclear cells
PMA: phorbol-12-myristate
QC: Quality Control
COVID-19: SARS-CoV-2 infection
TCR: T Cell Receptor
TNF: Tumor Necrosis Factor
