# Supplementary material for: A comprehensive longitudinal analysis of the cellular immune response specific to the spike protein in healthcare workers vaccinated against SARS-CoV-2– ORCHESTRA Project
Source: Front Immunol. 2025 Nov 25;16:1707449. doi: 10.3389/fimmu.2025.1707449 (PMC12685908; doi:10.3389/fimmu.2025.1707449)

Figure S1. Representative flow cytometry plots for analysis of activated CD4$^+$ and CD8$^+$ T-cells and their expression of effector cytokines. (**A)** PBMCs were gated on antigen-activated lymphocytes, single cells, live cells, to identify live CD3$^+$ T-cells. CD3$^+$ cells were further gated for CD4$^+$ and CD8$^+$ cells. Within the CD4$^+$ and CD8$^+$ T cell populations, activated CD4$^+$CD154$^+$ T cells, and CD45RO$^+$ surface markers were gated. Additionally, the expression of IFN-γ, IL-2, and TNF-α was analyzed within the CD4$^+$ and CD8$^+$ populations. **(B)** Representative smooth pseudocolour density plots including outliers for positive, spike-stimulated, and negative PBMC controls. PBMC: Peripheral blood mononuclear cells.

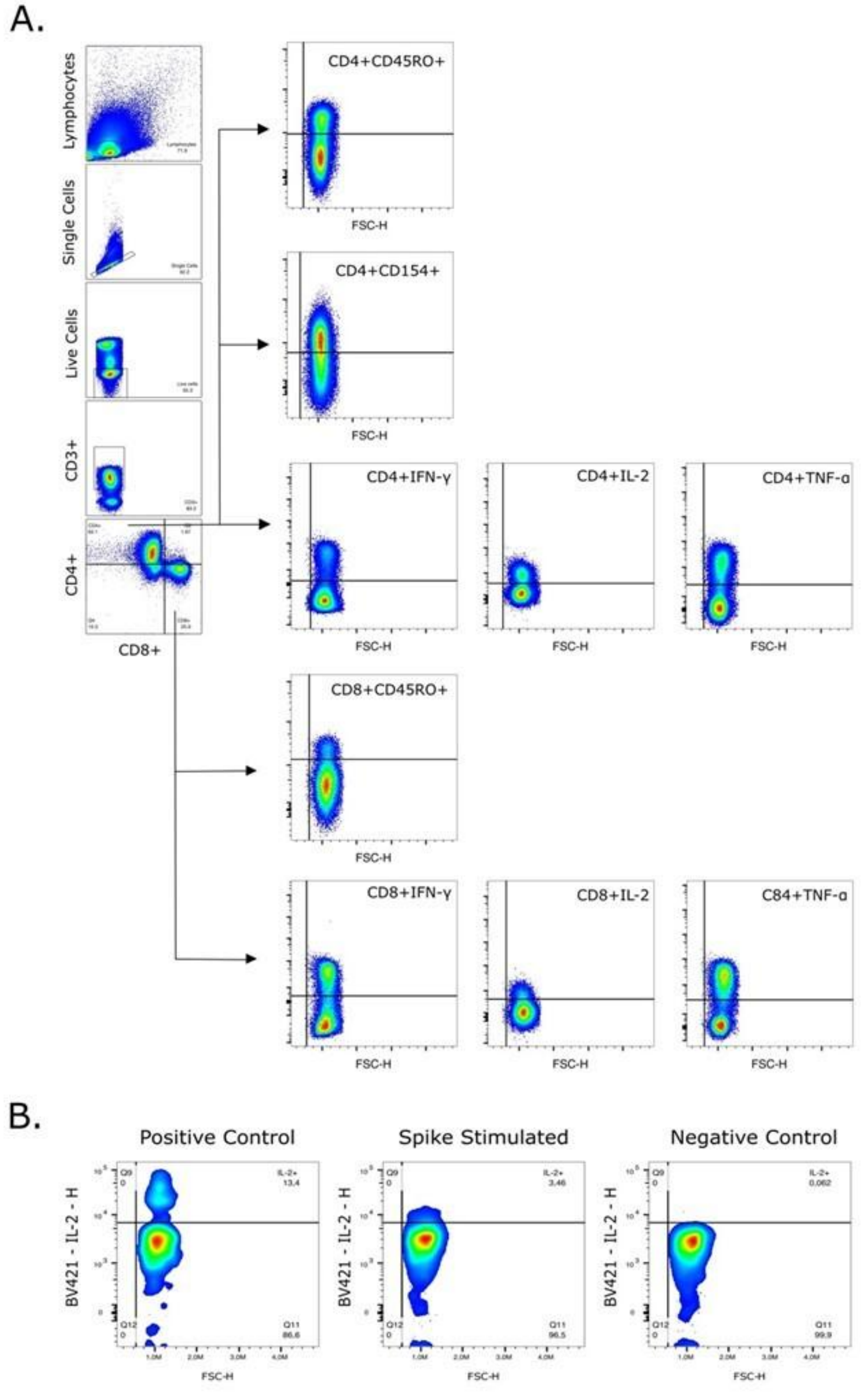

Supplement: Supplementary file 1 [file Image1.pdf]
