# Supplementary figures and images for: A comprehensive longitudinal analysis of the cellular immune response specific to the spike protein in healthcare workers vaccinated against SARS-CoV-2– ORCHESTRA Project
Source: Front Immunol. 2025 Nov 25;16:1707449. doi: 10.3389/fimmu.2025.1707449 (PMC12685908; doi:10.3389/fimmu.2025.1707449)

**Figure S2. Flow-chart showing criteria for samples exclusion (all participants and by Cohort)**

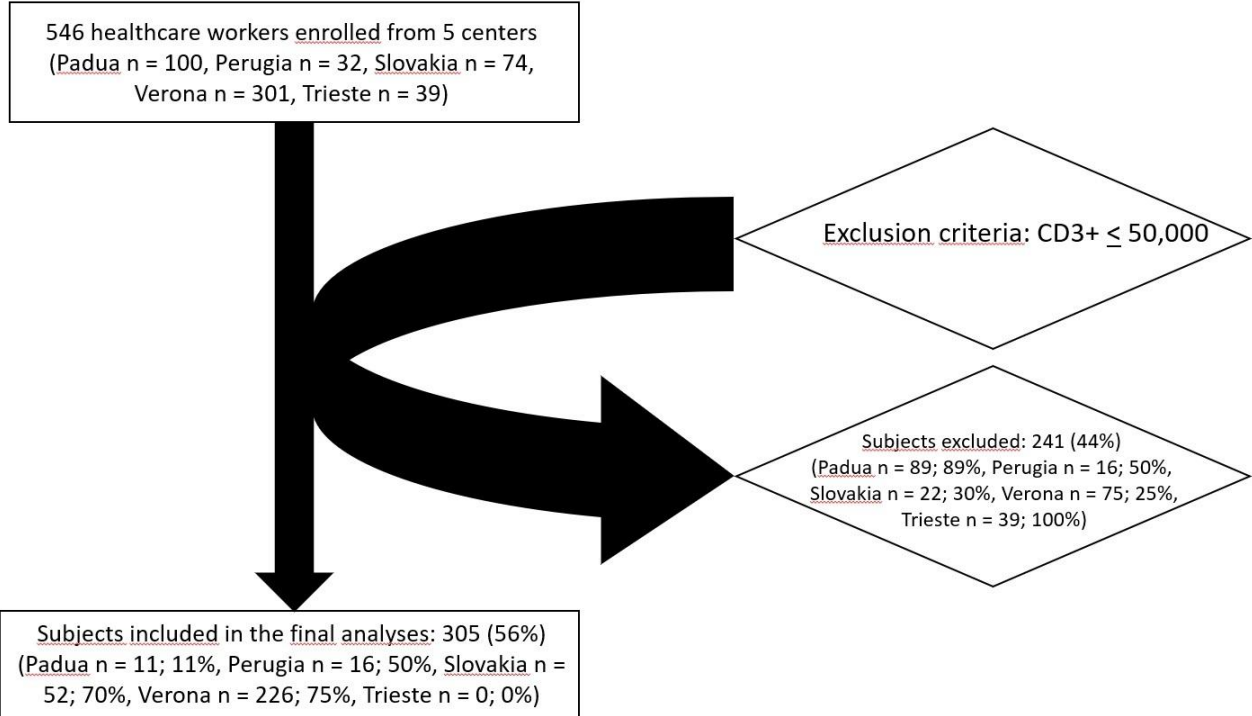

Supplement: Supplementary file 2 [file Image2.pdf]
