# Supplementary material for: A comprehensive longitudinal analysis of the cellular immune response specific to the spike protein in healthcare workers vaccinated against SARS-CoV-2– ORCHESTRA Project
Source: Front Immunol. 2025 Nov 25;16:1707449. doi: 10.3389/fimmu.2025.1707449 (PMC12685908; doi:10.3389/fimmu.2025.1707449)

Figure S3. Correlation between percentage of CD8 T cell response (cytokines and functional stage) and anti-S SARS-CoV-2 IgG titer in Verona and Perugia (panel left), Padua and Slovakia (panel right) cohorts.

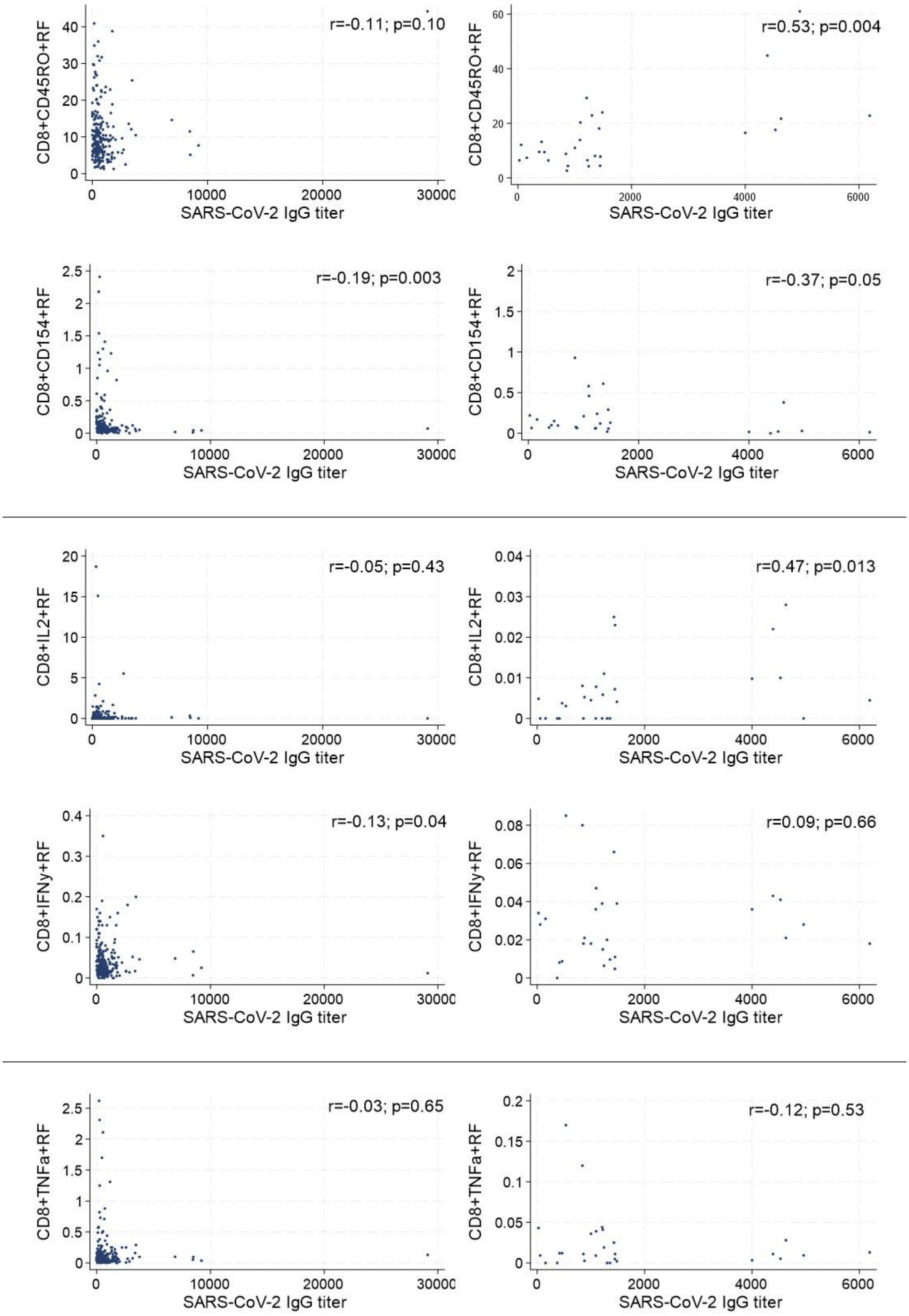

Supplement: Supplementary file 3 [file Image3.pdf]
