# Supplementary material for: A comprehensive longitudinal analysis of the cellular immune response specific to the spike protein in healthcare workers vaccinated against SARS-CoV-2– ORCHESTRA Project
Source: Front Immunol. 2025 Nov 25;16:1707449. doi: 10.3389/fimmu.2025.1707449 (PMC12685908; doi:10.3389/fimmu.2025.1707449)

Figure S4. Correlation between percentage of CD4 T cell response (cytokines and functional stage) and anti-S SARS-CoV-2 IgG titer in Verona and Perugia cohorts (panel left), and Padua and Slovakia cohorts (panel right).

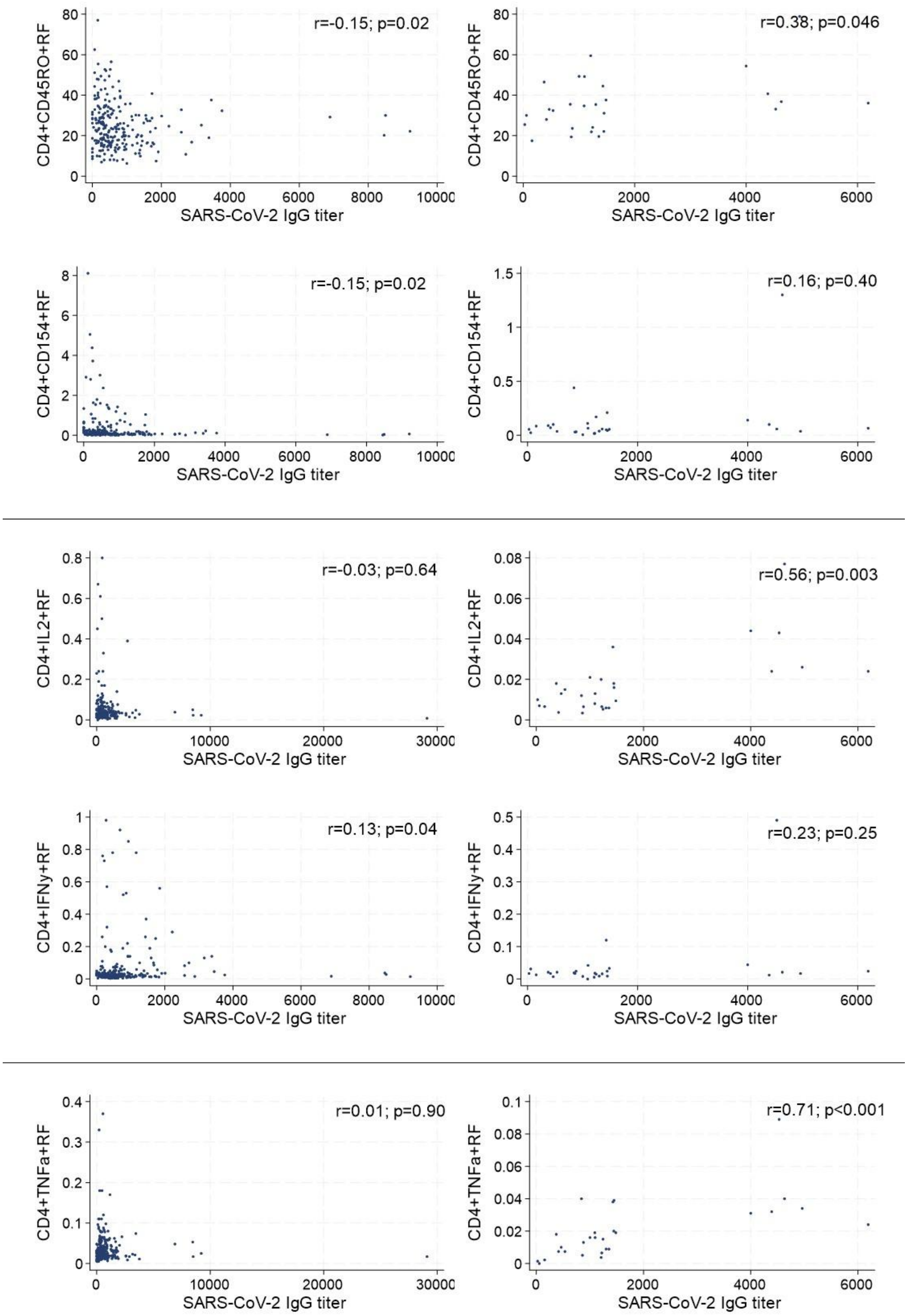

Supplement: Supplementary file 4 [file Image4.pdf]
