# Supplementary material for: A comprehensive longitudinal analysis of the cellular immune response specific to the spike protein in healthcare workers vaccinated against SARS-CoV-2– ORCHESTRA Project
Source: Front Immunol. 2025 Nov 25;16:1707449. doi: 10.3389/fimmu.2025.1707449 (PMC12685908; doi:10.3389/fimmu.2025.1707449)
Supplement: Supplementary file 5 [file Table1.docx]

**Supplementary material**

**Table S1. List of surface and intracellular staining antibodies**

| **Antibodies*** | **Clone** |
| --- | --- |
| **Surface Antibodies** | |
| APC anti-human CD154 | 24-31 |
| APC/Fire 750 anti-human CD3 | SK7 |
| PerCP/Cyanine5.5 anti-human CD45RO | UCHL1 |
| Brilliant Violet 570™ anti-human CD8a | RPA-T8 |
| FITC anti-human CD4 | RPA-T4 |
| **Intracellular antibodies** | |
| PE anti-human IFN-γ | 4S.B3 |
| PE/Cyanine7 anti-human TNF-α | MAb11 |
| Brilliant Violet 421 anti-human IL-2 | MQ1-17H12 |
